# Walking at work: Maximum gait speed is related to work ability in hospital nursing staff

**DOI:** 10.1002/1348-9585.12171

**Published:** 2020-10-12

**Authors:** Chad Aldridge, Victor Tringali, Robert Rhodes, Kohl Kershisnik, Debra Creditt, Jorge Gonzalez‐Mejia, Jose Lugo‐Vargas, Jean Eby

**Affiliations:** ^1^ Department of Physical Therapy University of Virginia Charlottesville VA USA; ^2^ Department of Public Health School of Medicine University of Virginia Charlottesville VA USA; ^3^ Hoo’s Well Employee Health University of Virginia Charlottesville VA USA; ^4^ School of Nursing University of Virginia Charlottesville VA USA

**Keywords:** gait speed, hospital, nurse, occupation, walking, work ability

## Abstract

**Objectives:**

Like the concept of work ability in occupational health, gait speed is a measure of general fitness and can predict functional decline and morbidity. This is especially important when our care‐takers, i.e. nurses, show decline in fitness and become care‐receivers. The study aims to describe the demographics of hospital nurses in the context of gait speed and work ability as well as to determine the association between them.

**Methods:**

Three‐hundred and twelve inpatient nurses and nursing assistants were sampled from a level 1 trauma and teaching hospital from several service lines and acuity levels. Spearman correlation tests were utilized to determine the relationship of gait speed and ratings of item 1 on the Work Ability Index (WAI) as well as Cochran‐Armitage test for linear trend of gait speed.

**Results:**

Maximum gait speed has a significant positive association with work ability with a Rho coefficient of 0.217 (*P* < .0001). Additionally, the linear trend test of gait speed tertiles was significant (*P* < .001) for work ability categories of Moderate to Poor (0‐7) and Good to Excellent (8‐10).

**Conclusions:**

Gait speed is correlated with the item 1 self‐rating of the WAI in hospital nursing staff. The 10‐m walk test is a practical and easy measure that can be utilized in occupational health. More research is required to validate gait speed in other occupational health populations and investigate gait speed changes and its interaction with the work environment longitudinally.

## 1 INTRODUCTION

In the medical field we are familiar with vital signs, like heart rate and blood pressure. One less well‐known vital sign is "Gait Speed.”^1^ It is a simple and objective measure that has been validated in diverse settings.^2^ Gait speed has been found to be a predictor of several health outcomes, including physical function, disability, and death in older adults.^3^, ^4^ The nursing work force is now moving into this “older adults” category, with the median age of nurses in the United States at 53 years old.^5^

In the realm of occupational health, the Work Ability Index (WAI)^6^ is a well‐tested and validated tool that measures an individual's lifestyle, work demands, and physical and mental function. The WAI is a questionnaire that is able to predict long‐term sickness, absence, and early retirement.^7^ It has been utilized in studying nursing populations.^8^, ^9^

Gait speed and work ability assess health status using different scales. Gait speed provides an objective measure of physical function and health at the moment of testing, while the WAI utilizes a respondent's overall perception of their own health and abilities. Both gait speed and work ability attempt to quantify how robust a person is. Measuring work ability along with gait speed may provide a more complete descriptor of an individual's ability to meet occupational demands, resiliency to work‐related injury, and risk of adverse health outcomes. This is especially important as the nursing work force, the universal care‐taker, ages and transitions to care‐receivers.

The aims of this study were (a) to describe the demographics and occupational characteristics of nursing staff in relation to gait speed and work ability and (b) to determine the association of gait speed and work ability.

## 2 METHODS

### 2.1 Design and setting

This was a cross‐sectional study of a hospital nursing staff cohort. It sampled inpatient nursing staff within an academic level 1 trauma medical center. Inpatient nursing staff included only the bedside nurses and the patient care assistants or technicians, “PCA”s or “PCT”s. Sampling included the following adult service lines: General Medicine, Cardiovascular and Thoracic, General Surgery and Trauma, Transplant, Urology, Neurology and Neurosurgery, and Orthopedics. Within services lines, if available, nursing staff of each acuity level were sampled, acute and critical care units. Each unit was sampled two times for each on‐coming shift. This medical center has a School of Nursing as well as relationship with a local community college nursing program.

### 2.2 Data collection

The study was approved by the Institutional Review Board at the University of Virginia, VA. Prior to any data collection, a study presentation was provided to the entire nursing management to explain its purpose and intent. Nursing administration permitted the study to be conducted with the stipulation that data collection minimizes interruption to nursing work flow and patient care.

Afterwards, each hospital's unit manager was also notified up to 2 weeks before data collection of that unit's nursing staff. The study's purpose and intent were presented directly to each unit's nursing staff during the 7 am and 7 pm “huddle” times when shifts exchange. Each interested individual received a verbal explanation of the study and its procedures, as well as potential future benefit for the individual and the nursing staff as a whole. Data were collected after verbal consent was obtained. Data were only collected for the oncoming shift within the first hour of the shift, e.g., the Day shift from 7 am to 8 am when participants filled out a data collection form that included demographic and work information. Variables queried included age, gender, height, weight, race/ethnicity, staff role, primary shift, years of experience, number of shifts worked consecutively, and a work ability item. The workability question was item 1 (WAS) from the WAI, which has been shown to be predictive of long‐term sickness and absence^10^ and feasible in occupational health research.^9^ Body mass index (BMI) was calculated from the reported height and weight measurements. Due to the short time window to collect data, objective measurement of gait speed was prioritized. In our opinion, BMI recorded in this manner was sufficient to check its expected relationship with gait speed.

After completing the data form, each participant performed the 10‐m walk test to measure gait speed. The study followed the 10‐m walk test protocol of Steffen and Seney.^11^ In brief, the 10‐m walk test had a 2‐m acceleration zone, 6‐m central zone, and a 2‐m deceleration zone. The participants were instructed to walk at their own “comfortable walking speed” from the first cone to the last cone. The time to ambulate the central zone distance was timed. The test was repeated for the maximum gait speed condition which instructed participants to “walk as fast as you safely can.” The test has been shown to be valid^12^, ^13^ and reliable.^13^

### 2.3 Statistical plan

All statistical analyses were conducted using SAS (version 9.4, SAS Institute Inc., Cary, NC, USA). Categorical data were summarized using counts and frequencies. Continuous data were either reported as means and standard deviations or as medians and interquartile ranges dependent upon the severity of deviations from normality. Kolomogorov‐Smirnov tests and quantile‐to‐quantile plots were utilized to determine normality of continuous variables. Because of non‐normality of the numeric variables, all comparisons utilized non‐parametric tests.

To determine the greatest association of WAS scores between self‐preferred and maximum gait speed, a Spearman's test of association was performed for both walking speed conditions. Also, participants who rated themselves with a WAS score of 0 to 7 were categorized as “Moderate to Poor” and those who had a rating of 8 or more were considered as “Good to Excellent” in terms of work ability. This classification scheme is consistent with other studies.^9^, ^14^ For gait speed measures, they were stratified in tertiles for both conditions. Binomial proportion tests were utilized to determine level of significance between work ability groups. Bonferroni adjustment was used to account for multiple comparisons among gait speed strata to reduce the chance of a Type I error.

## 3 RESULTS

### 3.1 Participants

Three‐hundred and seventy‐four nurses and PCA/PCTs volunteered for this study. Because of pregnancy status (n = 7) and data collection error of gait speed (n = 6), 13 individuals were excluded from the study, resulting in 361 participants. The sampling distribution among service lines was the following: General Medicine (100, 27.70%), Cardiovascular and Thoracic (78, 21.61%), General Surgery/Trauma/Transplant (38, 10.53%), Neurology and Neurosurgery (106, 29.36%), Orthopedics (27, 7.48%), and Float Pool (12, 3.32%). When accounting for the number of hospital units per service line, the sample sizes were similar and reflected the hospital nursing staff population.

There were additional missing data in several categories equaling 49 incomplete cases, resulting in 312 participants included in the statistical analyses. Missing data were found for age (12), experience (2), gender (6), BMI (21), and nursing role (8). Nurses accounted for 231 and PCA/PCTs totaled 81. The cohort was overwhelmingly female, and this included both the nurses (93.8%) and PCA/PCTs (86.8%), with a mean (SD) age of 32.28 ± 0.67 for nurses and 33.11 ± 1.32 for PCA/PCTs. Median (IQR) number of years of experience equaled 3.75 (1.5‐8) for nurses and 6.0 (3‐13) for PCA/PCTs.

Table 1 shows the demographic information of the study cohort. The four underweight individuals were grouped with normal weight participants. Over half (55.4%) of the nursing cohort was overweight or obese. They were also young with 54.8% between the ages of 18 and 29 where 51% had 4 years of experience or less.

**TABLE 1:** Cohort demographics

Total	312	
	n	%
Age categories		
18‐29	171	54.8
30‐39	72	23.1
40‐49	32	10.3
50‐59	29	9.3
60+	8	2.6
Experience		
0‐4	160	51.3
5‐9	72	23.1
10‐19	34	10.9
≥20	46	14.7
Gender		
Female	275	88.1
Male	37	11.9
Race		
African	39	12.5
Asian	7	2.2
Caucasian	225	72.1
Deferred	12	3.8
Hispanic	12	3.8
Other	17	5.4
BMI categories		
Normal weight	135	43.3
Overweight	82	26.3
Obese	91	29.2
Underweight	4	1.3
Nursing role		
Nurse	231	74.0
PCA/PCT	81	26.0
Acuity level		
Acute care	206	66.0
Critical care	106	34.0

Abbreviation: BMI, body mass index.

Gait speed achieved a significant spearman association with work ability. The maximum gait speed condition had a Rho coefficient of 0.217 (*P* < .0001), while the preferred gait speed condition had a coefficient of 0.109 (*P* = .041). Table 2 demonstrates the relationship between maximum gait speed and WAS rating. Overall, maximum gait speed was significantly associated with WAS rating (*P* = .003). Those in the lowest maximum gait speed tertile were significantly more likely to have a moderate‐to‐poor WAS rating (*P* = .015). In contrast, those in the highest maximum gait speed tertile were significantly more likely to have a good‐to‐excellent WAS rating (*P* = .014). Increasing maximum gait speed was associated with an increased likelihood of good‐to‐excellent WAS rating (*P* for trend < .001); see Table 2. We also investigated the increasing average maximum gait speed from poor workability to excellent workability. The standard errors had variable precision with the poor work ability category having the worst precision. This is likely due to a relative low amount of participants rating themselves as poor versus the other categories. Therefore, separating work ability into “Poor to Moderate” and “Good to Excellent” remained the best approach. There was no equivalent and consistent relationship observed between preferred gait speed and WAS rating.

**TABLE 2:** The relationship of work ability and gait speed

	Work ability groups				*P*‐value
	Moderate to poor		Good to excellent		
	n	%	n	%	
Total	83	26.6	229	73.4	
Max gait speed (m/s)					
<1.83	37	35.6	67	64.4	.015^a^
1.83‐2.04	27	26.7	74	73.3	.722
>2.04	19	17.8	88	82.2	.014^a^

Note

Max gait speed comparisons had the alpha adjusted to 0.016 to account for multiple comparisons.

^a^ Represents the comparisons that reached significance. Preferred gait speed did not achieve significance in any stratum between work Preferred gait speed did not achieve significance in any stratum between work ability groups.

**FIGURE 1:**
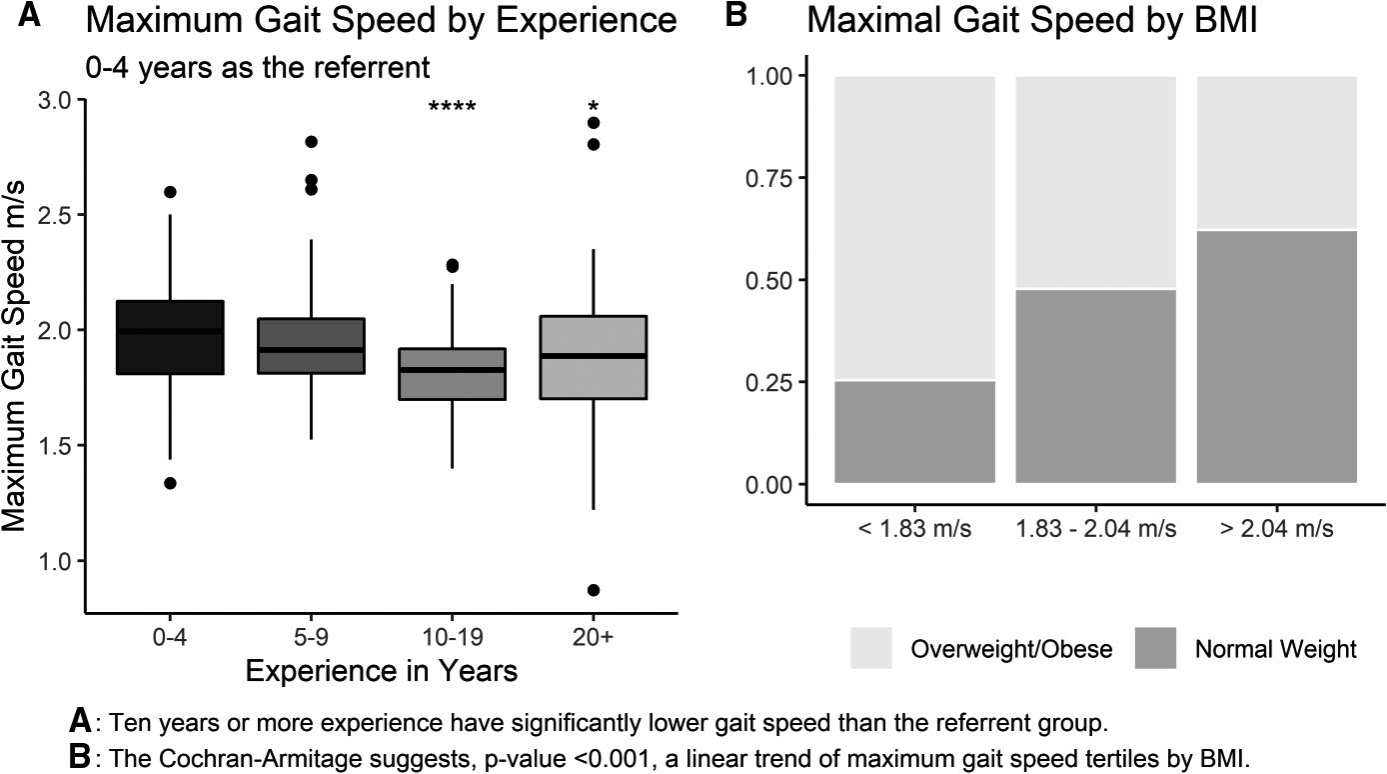
Relationship of Gait Speed to Experience (A) and BMI (B)

Secondary analysis revealed that BMI was negatively associated with gait speed and WAS ratings. BMI and maximum gait speed had a Rho coefficient of −0.369 (*P* < .001), while preferred gait speed had a smaller coefficient of −0.122 (*P* = .025). Maximum gait speed was also negatively associated with age (*P* = .005) and years of experience (*P* = .001), with respective Rho coefficients of −0.114 and −0.174. Maximum gait speed did not achieve significant differences among age ranges, but they did with ranges of experience in years, Figure 1A. The referent group with 0 to 4 years of experience had a significantly higher maximum gait speed compared to groups with 10 or more experience. The differences, however, were small at 0.16 and 0.10 m/s, respectively. The proportions of normal weight and overweight/obese participants stratified by maximal gait speed tertiles had significant trend with a *P*‐value of <.0001; Figure 1B visualizes the relationship.

BMI was further investigated as a possible effect measure modifier of maximal gait speed. A Chi‐square test was performed for work ability categories and maximal gait speed tertiles while stratified by BMI categories of (a) normal weight and (b) overweight or obese. The chi‐square test did not reach significance (*P* = .752) for the normal weight group, but it was significant in the overweight/obese group (*P* = .0378). Additionally, a test for trend also reached significance (*P* = .011) within the overweight/obese group.

## 4 DISCUSSION

This study's primary aim was to determine the relationship between walking speed and self‐reported work ability for hospital nursing staff. The results demonstrate a significant, but weak‐to‐fair, positive relationship between maximum gait speed and work ability. Nurses with faster maximal gait speeds are more likely to rate as Good or Excellent on the WAS. Conversely, nurses with the slowest gait speeds are more likely to rate Moderate or Poor on the WAS. Even though maximum gait speed had a weak to fair correlation with work ability, this has clinical importance as the concept of work ability is multifaceted consisting of physical and mental fitness, work place environment, and home life. Gait speed has the potential to be a meaningful predictor of work ability, which necessitates further research.

Unfortunately we found only one similar experiment in the literature that assessed work ability and physical function tests including gait speed. In opposition to this study's results, Padula et al. 2013 did not find a significant relationship between WAI scores and gait speed, for either self‐preferred or maximal conditions.^15^ Their study, however, had a much smaller sample size of 79 participants with only 12 participants self‐rating their work ability as poor. It is likely that the statistical analysis was underpowered. Their study also had a different design regarding data collection. Participants completed health and work ability forms outside of work time. Physical function measurements were taken at a separate location and time which does not ensure temporality with WAI scores. Our study controlled for time by collecting work ability and gait speed at the same time as well as before work‐related fatigue could become a confounder.

Overall this study shows that walking speed can be an important tool in employee health and wellness. Walking speed is a cost‐effective, easy to use, reliable, and valid measure.^3^, ^11^, ^16^, ^17^, ^18^ It has been shown to predict all‐cause mortality and cognitive decline in community older adults.^19^, ^20^, ^21^ Walking speed lends itself well to repeated measures for longitudinal occupational health studies. It is a measure of general fitness, while work ability itself, as described by Ilmarienen (2001), “the concept emphasizes that individual work ability is a process of human resources in relation to work.”^22^ He further describes that several factors affect work ability, especially “health and functional capacities” and the work environment. Since these factors change over time, studies have measured work ability over time.^23^, ^24^ Because of the demonstrated association between gait speed and work ability in this study, there may be similar value in longitudinal measurement of gait speed in an aging cohort in the context of the interaction of fitness and the work environment.

Gait speed can be a target for intervention. Van Abbema (2015) and colleagues concluded in a meta‐analysis on 25 studies that resistance training had a large effect on improving gait speed in older adults.^25^ A review by Coulter et al. also showed improvement of gait speed via physical therapy with increases in hip abductor and hip and knee extensor strength.^26^ This parallels the work of Van Der Krogt and Marjolein (2012) which investigated how sensitive is human gait to muscle weakness. They showed that human gait is sensitive to hip abductor, hip flexor, and plantar flexor weakness more than other muscle groups. Weakness in these muscle groups leads to gait impairment more readily than other lower extremity ones.^27^ Thus, it stands to reason that strength training of key muscle groups results in faster gait speed. In addition to strength training, Hortobayi et al (2015) found that coordination and multi‐modal training types also improve gait speed and health in older adults in their meta‐analysis^28^ of 42 studies. They further conclude that these interventions can slow or delay the onset of gait speed decline.

BMI is an additional factor to consider as an intervention. As this study shows, BMI has a significant negative association with gait speed and work ability. Rostamabadi et al also found the same association between BMI and work ability in a nursing cohort.^8^ The relationship between BMI and gait speed is expected, since BMI also influences gait mechanics,^29^ especially in the sagittal plane. In the same vein, increased energy cost to walking places individuals as young as 40 years old at a higher risk of a slow gait speed.^3^ Furthermore, a slow gait speed may predict mortality and cardiovascular disease (CVD). Veronese and colleagues^4^ via a systematic review and meta‐analysis demonstrated just that. They went on to recommend that it should be routinely utilized to screen people at risk for pre‐mature death and CVD in part because of its practicality as a measure. As a result, BMI is a reasonable target in an employee health intervention focusing on improving gait speed and work ability. This has great interest in the study of large populations, specifically, in the realm of occupational health.

Overall, this study had many strengths. It was conducted within a large hospital‐based nursing cohort. It sampled nursing staff from multiple service lines, such as Trauma and Neurology, as well as the acuity level of the units, acute versus critical care. Data collection occurred only within the first hour of the start of shift, which prevented work‐related fatigue from confounding walking speed and self‐rated work ability. Lastly, gait speed, and work ability were measured at the same time preventing recall bias.

### 4.1 Limitations

This medical center's population of nursing staff were younger than the national median age of 53 years,^30^ but similar to the study of ICU nurses by Rostamabadi et al.^8^ The two schools of nursing were the likely cause as newly graduated nurses with <2 years of experience made up a large proportion, 36.7%, of the nursing group. This limits the generalizability of this study to other hospital populations without local nursing schools or with older nursing populations. Furthermore, this nursing cohort may not generalize well to nursing populations in Community, Home Health, and Outpatient care settings. As a side effect of this cohort's age, >74% of participants were <40 years old. This is equivalent to experience with 74% having <10 years of experience. Because of the low numbers of older age and later careered participants, this study is not powered to sufficiently achieve an acceptable risk of a Type II error to control for age and experience in the statistical analysis. This is the case as well for the different service lines in which participants work. There is much heterogeneity within each service line. The nursing units within each service, however, had only 20 to 30 participants. Because of this, service lines and nursing units were not considered within the analysis.

## 5 CONCLUSION

Gait speed is correlated with the item one self‐rating of the WAI in hospital nursing staff. The 10‐m walk test is a practical measure that can be utilized as a tool to assess and be a target to improve employee health and wellness. This metric has potential to translate to a wide array of occupational health cohorts because of the minimal training and equipment required for reliable and valid measures. More research is required to further validate gait speed in other occupational health populations as well as investigate the relationship of gait speed and its interaction with the work environment longitudinally.

## DISCLOSURE

*Ethical approval:* The study received ethical approval from the Institutional Review board (IRB) of the University of Virginia. *Informed consent:* All participants provided informed verbal consent prior to participation. Written consent was waived by the IRB. *Registry and the registration no. of the study/trial:* N/A. *Animal studies:* N/A. *Conflict of interest:* Authors declare no conflict of interests for this article.

## AUTHOR CONTRIBUTIONS

CA conceived the idea and study design, analyzed the data, and led manuscript writing; VT assisted with study design, data interpretation, and manuscript writing; JE assisted with study design, data interpretation, and manuscript writing; RR collected data, audited data collection integrity, and manuscript writing; KK collected data, audited data collection integrity, and manuscript writing; DC collected data, nursing staff liaison, and manuscript writing; GMG collected data and manuscript writing; JLV collected data and manuscript writing.
